# Mercury Concentrations in Fish Jerky Snack Food: Marlin, Ahi, and Salmon

**DOI:** 10.1186/1476-069X-10-90

**Published:** 2011-10-11

**Authors:** Jane M Hightower, David L Brown

**Affiliations:** 1 Department of Medicine, California Pacific Medical Center, 2100 Webster Street Suite 418, San Francisco, California, 94115, USA; 2 Department of Geological and Environmental Sciences, Chico State University, Chico, CA, 95929, USA

**Keywords:** Ahi, fish, jerky, marlin, mercury, methylmercury, salmon

## Abstract

**Background:**

Dried meat and fish have served as an important durable nutrition source for humans for centuries. Because omega 3 fatty acids in fish are recognized as having antioxidant and anti inflammatory properties found to be beneficial for good health, many consumers are looking to fish as their main source of protein. Unfortunately, contaminants such as methylmercury can accumulate in some species of fish. The purpose of this research is to test commercially available fish jerky snack foods for mercury contamination.

**Methods:**

Fifteen bags of marlin jerky, three bags of ahi jerky, and three bags of salmon jerky were purchased from large retail stores in Hawaii and California, and directly from the proprietors' Internet websites. Five individual strips of jerky per bag were analyzed for a total of one hundred and five tests.

**Results:**

From the seventy-five marlin jerky samples, mercury concentration ranged from 0.052-28.17 μg/g, with an average of 5.53 μg/g, median 4.1 μg/g. Fifty-six (75%) marlin samples had mercury concentrations that exceeded the FDA's current mercury action level of 1.0 μg/g, while six samples had greater than 10 μg/g. Fifteen samples of ahi had mercury concentrations ranging from 0.09-0.55 μg/g, while mercury concentrations in fifteen salmon samples ranged from 0.030-0.17 μg/g.

**Conclusions:**

This study found that mercury concentrations in some fish jerky can often exceed the FDA's allowable mercury limit and could be a significant source of mercury exposure.

## Background

Humans have used dried meats, fish, and fruits as a portable nutrition source for centuries. Because omega 3 fatty acids in fish are recognized as having antioxidant and anti inflammatory properties found to be beneficial for good health, many consumers are looking to fish as their main source of protein. Fish products on the market can now be obtained as fresh, frozen, canned, smoked, or dried. Unfortunately, contaminants such as methylmercury can accumulate in some species of fish.

The United States Food and Drug Administration (FDA) regulates commercial fish and fisheries products, which includes jerky. Non-commercial fish are regulated by the United States Environmental Protection Agency (EPA). The current FDA and EPA joint advisory addresses "fish" and "shellfish," and their consumption for women who are pregnant, or want to become pregnant, nursing mothers, and young children. The advisory states they should not eat shark, swordfish, king mackerel, or tilefish because these species contain high levels of mercury. They are advised to eat up to twelve ounces (340 g) a week of a variety of fish and shellfish that are lower in mercury, such as shrimp, canned light tuna, salmon, Pollock, and catfish. They may also eat up to six ounces (170 g) of albacore ("white") tuna per week, as it can contain higher mercury levels [1].

The current allowable mercury level in commercial fish and fisheries products directed by the FDA is 1.0 μg/g. Of historic interest, this limit was originally derived form the conclusion of a court trial in 1977 with Anderson Seafood Inc, a swordfish proprietor, and the FDA. Data used to establish this level came from a massive poisoning episode that occurred in Iraq in 1971-2 from an organic mercury fungicide placed on grain, whereby the people used this grain for their daily bread consumption instead of for planting. In the court transcripts, there was much discussion as to whether a minimal clinical effect level could be established from the Iraqi data. Regardless, the court proceedings concluded that a minimal clinical effect level for humans was determined to be 400 μg/l whole blood. This allowed for the mercury limit set for fish of 0.5 μg/g to be raised to 1.0 μg/g, thus allowing fish greater than 0.5 μg/g to be sold in the commercial market [2,3].

The EPA still maintains a mercury limit set for non-commercial fish of 0.5 μg/g. In addition, since the FDA vs. Anderson Seafood Inc. trial, the EPA established a human tolerable limit for mercury exposure of 0.1 μg/kg body weight/day. For a 60 kg (132 lb) person, this would amount to an average of 42 μg of mercury allowed per week. Consistently consuming mercury at this amount is estimated to result in a blood mercury level of about 4-5 μg/l [4]. The FDA though, has not adopted the EPA's tolerable limit.

Mercury concentrations in some fish species can be quite variable, as mercury accumulates in the food chain. Longer lifespans, stronger predatory behavior, and greater number of miles traveled to diverse areas of exposure, are just a few reasons for this variability. Methylmercury is the predominant mercurial contaminant in fish. It can penetrate every cell in the body where it becomes strongly bound to tissues. No known cooking methods can remove it from fish tissue. As to fish products that have been dehydrated, the authors hypothesized that fish jerky would likely have mercury concentrations that were several times higher than found in fresh fish.

## Methods

Fifteen bags of marlin jerky, three bags of ahi tuna jerky, and three bags of salmon jerky were purchased from large retail stores in Hawaii and California, as well as from the proprietors' Internet websites. We analyzed five strips of jerky from each bag for a total of one hundred and five tests. Because strips from individual fish are combined in the preparation of the product, we assumed that each bag represented several different fish, possibly from the same catch. Total mercury concentrations (package weight) were performed by Microanalytical Systems Inc., Emeryville, CA. Their analysis is sensitive to a limit of detection of 0.002 μg/g, with an error margin of < 5% [5].

Two random samples each of ahi jerky and marlin jerky were also sent to the Wisconsin State Laboratory of Hygiene for confirmation of analysis. They use cold vapor atomic absorption. Their analysis is sensitive to a limit of detection of 0.004 μg/g, and an error margin < 10% [6].

Both labs obtained mercury concentrations from the jerky products without further dehydration or altering of water content.

Two blinded random samples of jerky taken from the packages marketed as marlin, and two from the ahi packages were sent for DNA identification to Fish DNA ID in St Augustine, Fla. They use a DNA barcoding technique to identify the species of fish for commercial and private interests [7].

## Results

**Figure 1:**
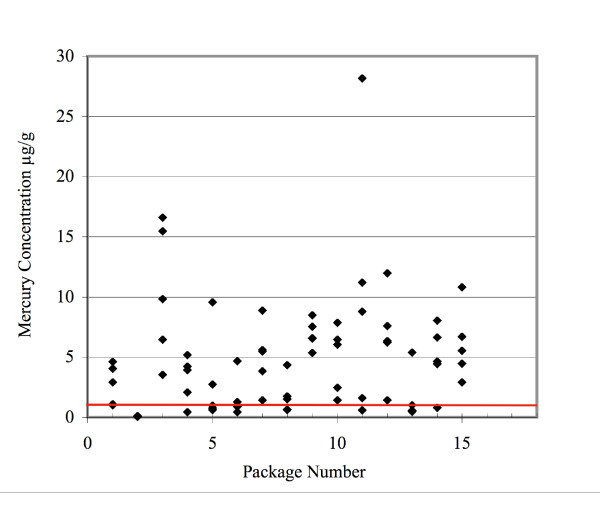
**Mercury Concentrations in Marlin Jerky**. The red line represents the FDA action limit of 1.0 μg/g.

From the seventy-five marlin jerky samples, mercury concentration ranged from 0.052-28.17 μg/g, (mean 5.53 μg/g). Fifty-six (75%) of the marlin test results were above the FDA's current mercury allowable level of 1.0 μg/g, while six samples had mercury concentrations greater than 10 μg/g (Figure 1). Sixty-seven (89%) of the marlin samples were above the EPA's 0.5 μg/g limit. Mercury concentrations in fifteen samples of ahi averaged 0.29 μg/g (range 0.09-0.547 μg/g), while concentrations in the fifteen salmon samples averaged 0.08 μg/g (range 0.030-0.174 μg/g).

The size of the jerky pieces, and the mercury concentrations between the bags were variable. Only bag number two had all five of its random sample test results less than the FDA standard for commercial fish of 1.0 μg/g. All other bags contained samples that tested greater than 4.0 μg/g. Bag number two also passed the EPA standard of 0.5 μg/g allowable limit for non-commercial fish.

Two marlin samples sent to the Wisconsin State Laboratory of Hygiene reported mercury concentrations of 9.3 μg/g and 9.7 μg/g. The two ahi sample results were 0.12 μg/g and 0.42 μg/g.

DNA analysis of jerky from packages labeled as ahi were identified as *Thunnus sp*, and matched with more genetic markers for ahi than bluefin or bigeye. The packages marketed as marlin were identified as blue marlin. The salmon jerky was not submitted for DNA analysis.

## Discussion

Despite a relatively small sample size, our findings indicate that some types of fish jerky can have a significant amount of mercury. FDA's analysis of thirty-four samples of fresh or frozen salmon found an average mercury concentration of 0.014 μg/g. In comparison, mercury concentrations in our fifteen salmon jerky samples were nearly six times higher averaging 0.078 μg/g. The FDA's database shows an average mercury concentration of 0.33 μg/g for eighty-seven samples of fresh or frozen ahi while the average concentration in the ahi jerky we sampled was essentially the same at 0.29 μg/g. This result is counterintuitive since the drying of fish is expected to increase the mercury concentration. The lower concentrations unexpectedly found in the ahi is possibly due to different sources of ahi or the selection of smaller, younger ahi for the preparation of jerky.

Since we did not further dehydrate the jerky specimens for an absolute dry weight concentration, the results could have some variability based on water content. The desiccation or smoking of fish will concentrate mercury. Regardless, the mercury concentrations obtained in this survey of packaged jerky is what the consumer would be ingesting at the point of sale.

In regard to marlin, the FDA analyzed sixteen samples from 1990-2002, but instead of reporting total mercury, only the methylmercury (MeHg) concentration was published. The percentage of MeHg to total mercury for this series of fish tested by the FDA was not reported. The average concentration of methylmercury in the FDA fresh marlin samples was 0.485 μg/g (range ND-1.079), compared to this paper's average total mercury of 5.53 μg/g. The type of marlin tested (striped, blue, black) by the FDA was not identified [8].

In 1972, mercury concentrations in blue and striped marlin were reported in the Proceedings of the International Billfish Symposium [9]. Fifty-six striped marlin, mostly caught from California coastal waters, ranged in weight from 25-105 kg (56-231.5 pounds), with a total mercury range of 0.03 to 2.1 μg/g. Blue marlin, which was caught in Hawaiian waters, weighed between 44-412 kg (96 to 906 pounds), and had mercury ranging from 0.7-7.86 μg/g. The livers of twenty-six blue marlin were also analyzed, and their mercury values ranged from 0.13-29.55 μg/g.

Blue marlin is among the largest marlin, and can reach 4 meters (fourteen feet) in length and weigh over 900 kg (1,980 pounds). The average size is 3.5 meters (eleven feet) and 90-182 kg (200 to 400 pounds). These fish are highly pelagic and greatly migratory, and can live more than twenty-five years [10].

The marlin jerky we tested came from 42 gram (1.5 ounce) to 198.6 gram (7-ounce) packages. Consumption of 42 grams of marlin jerky with an average mercury concentration of 5.5 μg/g identified in this paper would provide a mercury intake of 231 μg, while a 7-ounce package of jerky with this concentration would provide a mercury dose of about 1090 μg.

Although this product is marketed as a snack food, it is widely distributed and presumed popular in the state of Hawaii. It is unclear how widely distributed it is in other geographic areas where marlin consumption also occurs, such as along the gulf coast and in southern states, and unincorporated territories of the United States such as the Marianas Islands and American Samoa.

## Conclusions

This study found that mercury concentrations in some fish jerky products can exceed the FDA's allowable mercury limit for fish products and could be a significant source of mercury exposure for the consumer. Since fish jerky is readily available in some areas and may be perceived as a healthy snack food, adequate monitoring, labeling and consumer advice is needed to reduce methylmercury exposure, which is known to pose serious developmental risks.

## List of abbreviations

RfD: Reference Dose; FDA: United States Food and Drug Administration; EPA: United States Environmental Protection Agency; Kg: Kilogram; μg: microgram; MeHg: Methylmercury; NFL: National Food Lab.

## Competing interests

The authors declare that they have no competing interests.

## Authors' contributions

JH was responsible for concept, design, data collection, analysis and interpretation of data, and writing of this manuscript. DB was responsible for data collection, analysis and interpretation of data, and writing of this manuscript. All authors read and approved the final document.
